# Exosomes in hypertension: mechanisms, biomarkers, and therapeutic opportunities

**DOI:** 10.3389/fimmu.2026.1830650

**Published:** 2026-09-22

**Authors:** Dexiu Li, Dongyue Ma, Ziyi Qiu, Lin Zhao, Liying Zheng, Mei Xue

**Affiliations:** 1 Xiyuan Hospital, China Academy of Chinese Medical Sciences, Beijing, China; 2 National Clinical Research Center for Chinese Medicine Cardiology, Beijing, China; 3 Xiyuan Hospital of Clinical Medical College of Beijing University of Chinese Medicine, Beijing, China

**Keywords:** exosomes, hypertension, immunomodulation, inflammation, microRNAs, RAAS, vascular remodeling

## Abstract

Exosomes have emerged as important mediators in the pathophysiology of hypertension, facilitating intercellular communication through the transfer of bioactive molecules such as microRNAs. This review synthesizes current evidence on the multifaceted roles of exosomes in hypertensive vascular damage, highlighting their involvement in endothelial dysfunction, vascular smooth muscle cell phenotypic switching, renin–angiotensin–aldosterone system modulation, and renal ion transport regulation. Circulating exosomal microRNAs are identified as central drivers of inflammation and vascular remodeling, while urinary exosomes offer non-invasive insights into renal sodium handling and early hypertensive injury. Conversely, mesenchymal stem cell-derived exosomes demonstrate considerable vasoprotective and immunomodulatory properties, positioning them as therapeutic candidates. We further discuss emerging engineering strategies, both endogenous and exogenous, that aim to enhance exosomal targeting, cargo delivery, and therapeutic efficacy. Despite progress, challenges related to biological heterogeneity and long-term safety remain barriers to clinical translation. This review underscores the dual potential of exosomes as diagnostic biomarkers and therapeutic platforms, while outlining directions for future research toward clinical implementation in hypertension management.

## 1 Introduction

Hypertension remains a leading global risk factor for cardiovascular morbidity and mortality. While conventional therapies targeting neurohormonal pathways, such as the renin-angiotensin-aldosterone system (RAAS), have significantly improved blood pressure control, a substantial proportion of patients continue to face persistent clinical challenges, including treatment resistance and progressive target-organ damage. This unmet clinical need underscores the importance of elucidating novel pathophysiological mechanisms and identifying robust biomarkers to refine risk stratification and therapeutic intervention.

Extracellular vesicles (EVs), specifically exosomes (30–150 nm), have emerged as critical mediators of intercellular communication in hypertension (1, 2). Originating from the endosomal pathway, exosomes encapsulate a diverse repertoire of bioactive cargo, including microRNAs (miRNAs), long non-coding RNAs, messenger RNAs, proteins, and lipids, within a stable lipid bilayer (3). This structural integrity in biofluids like plasma and urine facilitates long-distance signaling between disparate organ systems. Accumulating evidence suggests that exosomes from pathologically altered cells can actively propagate hypertensive signals. For instance, exosomes isolated from monocrotaline-induced models can trigger pulmonary hypertension in healthy recipients, a phenomenon largely driven by the enrichment of pro-proliferative and pro-inflammatory miRNAs, such as miR-19b and miR-14 (4). Hypertensive conditions also stimulate increased exosomal release from vascular endothelial cells, contributing to essential pathological processes such as vascular smooth muscle cell (VSMC) phenotypic switching and endothelial–mesenchymal transition (5). Conversely, exosomes derived from mesenchymal stromal cells (MSCs) exhibit significant vasculoprotective potential, attenuating experimental hypertension through the delivery of anti-inflammatory miRNAs like miR-124 (4, 6).

Beyond their mechanistic roles, exosomes are promising diagnostic and theragnostic candidates. Specific exosomal miRNA signatures, such as the upregulation of miR-1246 and the downregulation of miR-184, hsa-miR-432-5p, and hsa-miR-1-3p, have been identified as high-value markers in hypertensive cohorts (7). Furthermore, urinary exosomes offer a non-invasive window into renal pathology; for example, reduced levels of urinary exosomal miR-26a-5p and miR-146a have been linked to early hypertensive kidney injury (8, 9). Consequently, exosomes are increasingly viewed as a novel class of biomarkers for disease classification and longitudinal monitoring (**Figure 1**).

**Figure 1:**
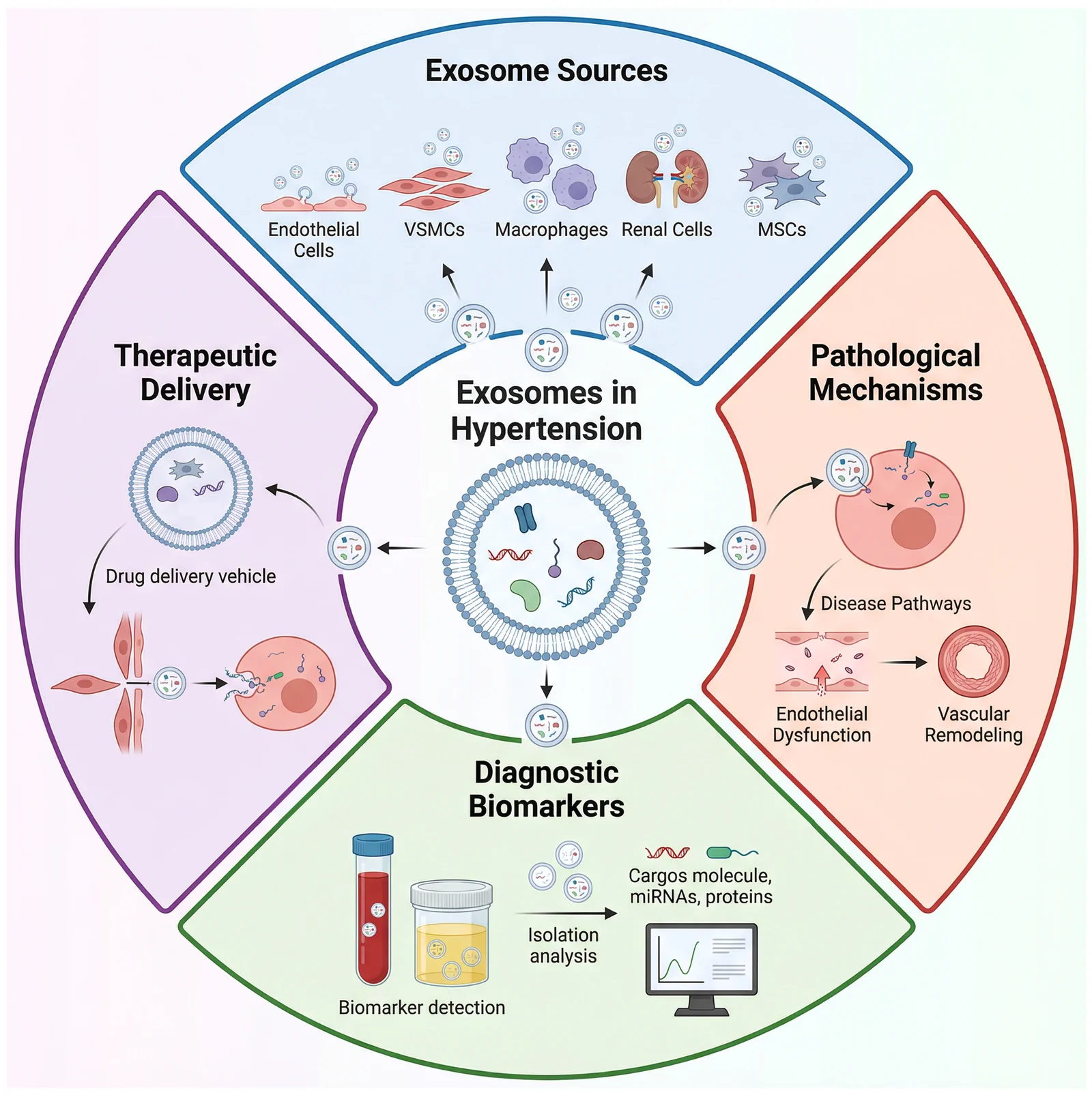
The sources of exosomes and their applications in hypertension. Exosomes, secreted by multiple cell types, participate in the pathogenesis of hypertension by mediating vascular remodeling and other pathological processes, and serve as diagnostic biomarkers and therapeutic delivery vehicles for hypertension management.

This review summarizes current evidence on the roles of exosomes in hypertension. We explore the distinct roles of circulating, urinary, and stem cell-derived exosomes, evaluating their potential as biomarkers and therapeutic agents to highlight translational opportunities in this rapidly evolving field.

## 2 Search strategy

This mini review is a narrative review. A comprehensive literature search was conducted using the online databases PubMed, Web of Science, and China National Knowledge Infrastructure (CNKI). The search strategy employed combinations of the keywords, such as “exosome”, “exosomal”, “exosomic”, “extracellular vesicle”, “microRNA”, “hypertension”, “high blood pressure”, and “vascular remodeling”. The retrieved articles were screened by title, abstract, and full text to exclude those not aligned with the scope of this review. The search primarily covered publications from 2015 to 2025, with additional relevant articles outside this range included as appropriate.

## 3 Exosome-mediated mechanisms implicated in hypertension

Exosomes are secreted by a diverse array of cell types, including hematopoietic cells, fibroblasts, vascular endothelial cells, and VSMCs. They are ubiquitously distributed across biological fluids, such as blood, urine, cerebrospinal fluid, amniotic fluid, semen, and bronchoalveolar lavage fluid (10). Emerging evidence indicates that exosomes actively mediate intricate crosstalk among vascular, immune, and neuroendocrine systems, contributing to the initiation and progression of hypertension. Specifically, exosomal miRNAs have been implicated in cardinal pathogenic processes, including endothelial dysfunction, vascular remodeling, chronic inflammation, and dysregulation of neurohumoral signaling (**Figure 2**). **Table 1** compiles the role of exosome-derived mirnas in hypertension. By facilitating the horizontal transfer of bioactive cargo between cells, these miRNAs serve as pivotal messengers in the propagation of hypertensive signals. The following sections provide a detailed overview of the specific roles played by exosomal miRNAs in the pathogenesis of hypertension.

**Figure 2:**
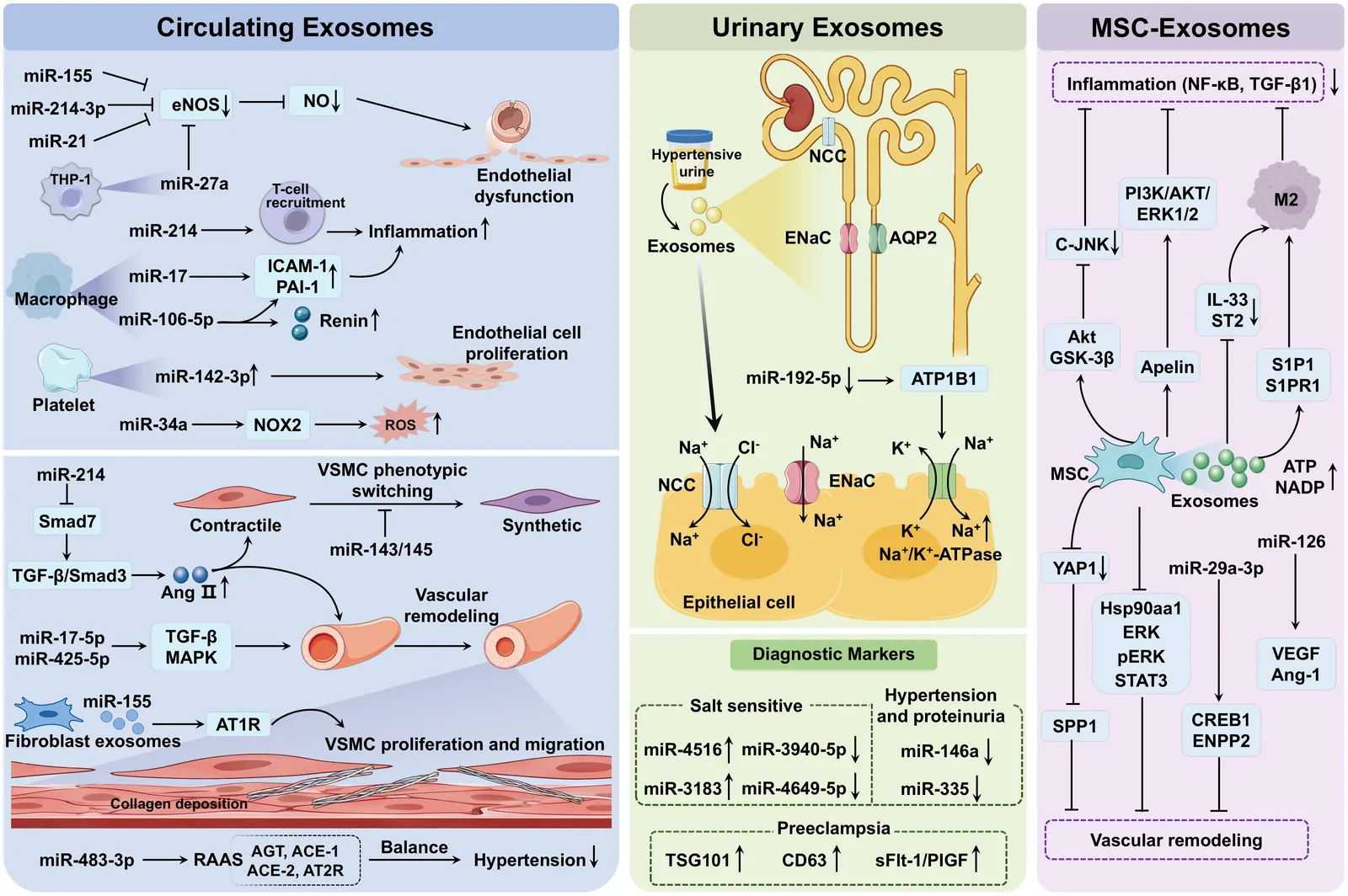
Exosomes in hypertension: pathophysiological roles and regulatory mechanisms. Circulating, urinary, and mesenchymal stem cell–derived exosomes carry specific miRNAs and functional proteins, which critically regulate endothelial dysfunction, VSMC phenotypic switching, paracrine activation of the RAAS, and renal sodium–water transport, thereby contributing to the initiation and progression of hypertension.

**Table 1:** Role of exosome-derived miRNAs in hypertension.

miRNAs	Experimental types	Models	Mechanisms	Signal molecules	References
miR-155	*In vitro*, Human samples	HUVECs, Human internal mammary artery rings	Endothelial dysfunction	↓:eNOS, NO	(11)
miR-155	*In vivo* and vitro	Spontaneously hypertensive rats, HUVECs	Endothelial dysfunction	↓: OPG, NO, eNOS ↑:RANK/RANKL, NF-κB, TNF-α, IL-6	(12)
miR-214	*In vivo* and vitro, Human samples	Ang II, miR-214^−^/^−^, and RAG1^−^/^−^ mice; CD3^+^ T cells; Hypertensive patients	Endothelial dysfunction, Fibrosis, Oxidative Stress, Inflammation	↑:CCR1/2/4/5/6, CXCR3, IL-17, TNF-α, IL-9, IFN-γ, TGF-β, Nox2, Col1a1/Col3a1/Col5a1, Pulse wave velocity ↓: NO	(13)
miR-214-3p	*In vivo*, Human samples	Dahl salt-sensitive rats, Human kidney biopsy sample	Endothelial dysfunction, Inflammation, Fibrosis	↓: eNOS ↑: TNF-α, MCP1, IL-6, α-SMA, Col4a, Albuminuria	(14)
miR-34a	*In vitro*, Human samples	HUVECs, Hypertensive patients	Endothelial dysfunction	↓: TIGF2, G1/S phase transition	(18)
miR-146a-5p	*In vivo*	Spontaneously hypertensive rats	Oxidative Stress, Inflammation	↓: NF-κB ↑: SOD2	(19)
miR-142-3p	*In vivo* and vitro	Renal hypertensive rats, Rat aortic endothelial cells	Endothelial cell proliferation, Platelet activation, Apoptosis	↓: BCLAF1 ↑: BCL2/Bax	(21)
miR-106b-5p	*In vivo* and vitro	KODMAC mice, Macrophages	Endoplasmic reticulum stress, Inflammation	↓: E2f1, Pde3b ↑: cAMP, p-CREB, PPARγ, Renin	(22)
miR-27a	*In vivo* and vitro	SD rats, HUVECs, THP-1 cells, HEK-293 cells	Inflammation, Vasodilatation	↓: eNOS; Ang- (1–7)	(23)
miR-17	*In vivo* and vitro	Ang II-induced hypertensive rats, THP-1 cells, HCAECs	Inflammation	↓: ICAM-1, PAI-1	(24)
miR-143/145	*In vivo* and vitro	HUVECs, HAECs, HASMCs, ApoE^−/−^ mice	VSMCs phenotype, Atherosclerosis	↓: ELK1, KLF4, CAMK2d, SSH2, CFL1, PHACTR4	(29)
	*In vivo*	miR-143/145^-/-^ mice	VSMCs phenotype	↓: ACE-1	(30)
miR-214	*In vivo*	miR-214 KO mice, Ang-II-induced hypertensive rats	VSMCs phenotype, Vascular Remodeling, Inflammation	↓: Smad7 ↑: TGF-β, Smad3, IL-6, MCP-1, TNF-α	(33)
miR-155-5p	*In vivo* and vitro	Spontaneously hypertensive rats, VSMCs	Vascular Remodeling, VSMCs proliferation	↓: ACE, Ang II, AT_1_R, PCNA	(34)
miR-483-3p	*In vitro*	HASMCs, RASMCs, HEK-293 cells, mice heart	RAAS, Vascular Remodeling	↓: AGT, ACE-1, ACE-2, AT_2_R	(35)
miR-192-5p	*In vitro*; Human Samples	Human Kidney Biopsy Samples, Salt-sensitive and congenic SS.13^BN26^ rats, miR-192 KO mice	Renal tubular reabsorption	↓: ATP1B1	(43)
miR-126	*In vivo* and vitro	HUVECs, C57BL/6 mice	Angiogenesis, Endothelial cell proliferation and migration	↓: PIK3R2 ↑: PI3K, Akt; VEGF, Ang-I	(47)
miR-1246	Human plasma samples	Patients with pulmonary arterial hypertension	Pulmonary artery smooth muscle proliferation, Vascular remodeling	↑: Ras, MAPK, Rap1	(57)
miR-26a	*In vivo* and vitro	Spontaneously hypertensive rats, Ang-II-induced hypertensive mice, Rat thoracic aorta VSMC	VSMCs proliferation, Vascular remodeling, Fibrosis	↓: Smad4, CTGF, EZH2 ↑: p21	(58)
miR-26a-5p	*In vitro*, Human plasma samples	Patients with pulmonary arterial hypertension, Human pulmonary artery endothelial cells	Endothelial proliferation, Vascular remodeling	↑: NF-κB, VEGF	(59)
miR-126-3p	*In vitro*, Human samples	Hypertensive patients, HUVECs	Inflammation, Vascular remodeling, Metabolic disorders	↓: HIF-1α, VEGF, TNF-α, IL-6, CRP	(60)
miR-184	Human plasma samples	Patients with essential hypertension	Inflammation, Vascular remodeling, Cellular senescence, Hormone synthesis and secretion	↓: PI3K/Akt	(7)
miR-432-5p	*In vitro*	Human pulmonary artery smooth muscle cells, Pulmonary microvascular endothelial cells, Perithelial cells	VSMCs proliferation, Vascular remodeling	↓: DEPDC1, POLR2D	(61)
miR-1-3p	*In vivo* and vitro	Hypoxic pulmonary hypertension mice, Human pulmonary artery smooth muscle cells	VSMCs proliferation and migration, Vascular remodeling	↓: SphK1, S1P	(62)
miR-222-3p	*In vitro*, Human samples	Preeclampsia patients, Human extravillous trophoblast cells	Trophoblast cells proliferation and migration, Vascular remodeling	↓: STMN1, MMP-2/9	(63)
miR-191-5p	Human samples	Hypertensive patients, Hypertension and metabolic syndrome patients, Hypertension and chronic kidney disease patients	Inflammation, Renal function, Vascular remodeling	\	(64)
	*In vivo* and vitro, Human samples	Placental tissue from patients with early-onset preeclampsia, Human umbilical cord MSCs, Thp-1 macrophages, Early-onset preeclampsia mice	Macrophages polarization, Trophoblast cells migration	↓: CDK6 ↑: M2 polarization, PDGF-AB, PDGFR-β, PI3K-AKT-mTOR	(65)

### 3.1 Circulating exosomes

#### 3.1.1 Endothelial dysfunction and immune inflammation

Hypertension is fundamentally characterized by chronic low-grade inflammation, with endothelial dysfunction serving as a primary driver of vascular impairment. Emerging evidence suggests that exosome-derived miRNAs constitute a key mechanistic link between inflammatory activation and endothelial injury, primarily through disruption of the endothelial nitric oxide synthase (eNOS)/nitric oxide (NO) signaling axis.

Several exosomal miRNAs directly impair the eNOS/NO pathway to promote endothelial dysfunction. miR-155 serves as a potent negative regulator of eNOS expression; its overexpression suppresses NO production and activates vascular endothelial growth factor (VEGF)-related signaling, thereby exacerbating endothelial dysfunction (11). In spontaneously hypertensive rats (SHR), elevated miR-155 expression is closely correlated with enhanced vascular inflammation, and this phenotype can be partially reversed by L-amlodipine treatment via the inhibition of the miR-155 axis and modulation of RANK/RANKL/OPG pathway (12). Perivascular adipose tissue–derived miR-214 promotes T cell recruitment and activates pro-fibrotic and chemotactic signaling, thereby linking immune cell infiltration with structural vascular remodeling. miR-214 knockout significantly reduces T cell infiltration, inhibits vascular remodeling, and stabilizes blood pressure, in part through eNOS targeting (13, 14).

miR-21 is widely expressed in vascular and immune cells and is highly sensitive to shear and mechanical stress (15). Circulating levels are elevated in hypertension patients and correlate positively with 24-hour ambulatory blood pressure and carotid intima–media thickness, while associating inversely with NO bioavailability (16, 17). In parallel, miR-34a is upregulated in essential hypertension and, by targeting TGIF2, inhibits endothelial cell proliferation and promotes apoptosis, thereby exacerbating endothelial injury (18).

In addition to directly damaging endothelial cells, exosomal miRNAs also participate in the regulation of immune-inflammatory balance. In hypertensive states, miR-146a-5p appears to act as a compensatory anti-inflammatory regulator induced by myocardial inflammation and endothelial activation (19). This protective response can be attenuated in sedentary individuals, where lower miR-146a levels, alongside elevated miR-34a levels, correlate with higher blood pressure, potentially reflecting exhaustion of anti-inflammatory compensation (20).

Intercellular transfer of exosomal cargo further amplifies these processes. In renal hypertension rats, platelet-derived exosomes deliver miR-142-3p to endothelial cells, promoting endothelial cell proliferation via BCLAF1 suppression and Bcl-2/Bax upregulation (21). Macrophage-derived exosomal miR-106b-5p, induced by endoplasmic reticulum stress, stimulates renin production through inhibiting E2F1 and phosphodiesterase 3B (22), exemplifying cross-talk between immune activation and neurohormonal control. Similarly, THP-1–derived exosomal miR-27a suppresses endothelial Mas receptor expression and eNOS phosphorylation, impairing Ang- (1–7)–mediated vasodilation and elevating blood pressure (23). Conversely, Ang II stimulation reduces exosomal miR-17 levels, a microRNA that normally restrains endothelial inflammation; its downregulation increases the expression of ICAM-1 and plasminogen activator inhibitor-1 (PAI-1), further amplifying endothelial inflammation (24).

In summary, these findings indicate that exosomal miRNAs participate in both initiating endothelial dysfunction and sustaining immune-inflammatory amplification. The direction and magnitude of these effects vary with cellular origin and disease context, and the extent to which these observations translate to human essential hypertension warrants continued investigation.

#### 3.1.2 VSMC phenotypic switching, vascular remodeling, and the RAAS

Vascular remodeling is a cornerstone of hypertensive progression, fundamentally driven by the phenotypic switching of VSMCs from a contractile to a synthetic state. This transition facilitates abnormal proliferation, migration, and extracellular matrix deposition, leading to arterial wall thickening and reduced compliance (25). Intraperitoneal injection of circulating exosomes from SHR into normotensive rats is sufficient to induce systemic hypertension, aortic thickening, and perivascular fibrosis, providing direct *in vivo* evidence that exosome-mediated intercellular communication is not merely associative but functionally sufficient to drive hypertensive vascular remodeling (26).

Mechanistically, exosomes can activate the RAAS through paracrine signaling. Exosomes released from adventitial fibroblasts of SHR are enriched in angiotensin-converting enzyme (ACE) and can be internalized by VSMCs, thus enhancing local Ang II generation and activating angiotensin type 1 receptor (AT_1_R) signaling, which in turn promotes VSMC migration and structural remodeling. However, inhibiting exosome release or blocking the ACE/AT_1_R axis can markedly attenuate these effects (27).

Exosomes can also deliver pathogenic cargos that exacerbate vascular injury. For instance, in pulmonary hypertension models, exosomal HCG20 promotes VSMC proliferation while inducing endothelial pyroptosis and endothelial–mesenchymal transition, further aggravating vascular remodeling (28). Specific exosomal miRNAs serve as rheostats for VSMC homeostasis. The miR-143/145 cluster, typically transferred from endothelial cells to VSMCs under laminar shear stress, is essential for maintaining the contractile phenotype (29). Deletion of this cluster promotes VSMC transition to the synthetic phenotype, accompanied by ACE-1 upregulation, neointimal formation, and elevation of blood pressure (30). Conversely, twenty-three exosomal miRNAs are markedly upregulated in the plasma of SHR, among which miR-17-5p and miR-425-5p participate in inflammation and vascular remodeling through TGF-β/MAPK pathways (31).

The interaction between RAAS effectors and miRNAs further amplifies vascular damage. Ang II functions not only as a potent vasoconstrictor but also as a pro-remodeling mediator that stimulates VSMC proliferation and induces the release of pro-inflammatory cytokines such as IL-6 and PAI-1 (32). Within this environment, miR-214 enhances TGF-β/Smad3 signaling by suppressing Smad7, and then promotes Ang II-induced medial thickening, whereas inhibition of miR-214 alleviates hypertension and reverses remodeling in mice (33). Ren et al. demonstrated that EVs derived from SHR rats are enriched in ACE but deficient in miR-155-5p, a combination that promotes VSMC proliferation, aggravates VSMC remodeling, leading to elevated blood pressure. In contrast, miR-155-5p overexpression or infusion of EVs containing miR-155-5P can inhibit ACE/Ang II/PCNA pathway, significantly lower blood pressure, and attenuate VSMC remodeling (34). Additionally, miR-483-3p has emerged as a systemic regulator of RAAS homeostasis by targeting multiple components, including AGT, ACE-1, ACE-2, and AT_2_R (35).

Collectively, these findings position exosomal miRNAs as central mediators of the crosstalk between the systemic RAAS and local vascular architecture. In a word, exosomal miRNAs act as key modulators linking RAAS activation to VSMC phenotypic switching, although their translational relevance in human hypertension requires further validation.

### 3.2 Urinary exosomes

Urinary exosomes provide a non-invasive “liquid biopsy” of renal physiology. They originate from various nephron segments and encapsulate segment-specific transporters, including sodium-hydrogen exchanger 3 (NHE3), aquaporin 2 (AQP2), sodium–chloride cotransporter (NCC), and the epithelial sodium channel (ENaC). The composition of urinary exosomes is intricately regulated by aldosterone and the RAAS, making them sensitive indicators of renal ion transport, salt sensitivity, and inflammatory status (36, 37).

RAAS activation can modulate NCC and ENaC expression in urinary exosomes (38), while aldosterone promotes distal tubular Na^+^ reabsorption by activating ENaC. In parallel, extracellular K^+^ concentration serves as a key physiological regulator of NCC abundance and phosphorylation, underscoring the integrated control of sodium–potassium balance (39). In salt-sensitive hypertensive models, both NCC expression and its phosphorylation level in urinary exosomes are pronouncedly elevated, a phenomenon that is associated with enhanced NaCl reabsorption, sequentially contributing to blood pressure elevation (40). Moreover, NCC and prostasin exhibit diurnal rhythms that parallel antidiuretic hormone secretion and AQP2 expression (41). Together, these observations support a functional association between urinary exosomal cargo and systemic blood pressure homeostasis, although mechanistic inference remains indirect.

Urinary exosomal miRNAs provide novel molecular insights into renal mechanisms and phenotypic heterogeneity in hypertension. Gildea et al. identified 194 miRNAs in urinary exosomes from hypertensive patients, among which 45 exhibited differentially expression across salt-sensitive, salt-resistant, and inverse salt-sensitive phenotypes. For example, miR-4516 is upregulated in salt-sensitive patients but downregulated in individuals with inverse salt sensitivity, while miR-3183 is elevated in both salt-sensitive and inverse salt-sensitive groups. In contrast, miR-3940-5p and miR-4649-5p are reduced in these same groups (42). Downregulation of miR-192-5p in hypertensive patients and salt-sensitive rat kidneys is associated with elevated blood pressure, mechanistically, miR-192-5p may exert protective effects by regulating Na^+^/K^+^-ATPase activity through inhibition of ATP1B1 (43). However, whether these miRNA alterations are drivers of hypertension or secondary adaptations to altered renal hemodynamics remains to be fully elucidated.

In hypertensive patients with proteinuria, urinary exosomal miR-146a and miR-335 levels are substantially decreased (9), indicating a potential association with early renal injury, although their diagnostic specificity requires further validation in larger cohorts. In preeclamptic patients, both urinary exosome concentration and the expression of TSG101 and CD63 are significantly increased,and the urinary exosomal sFlt-1/PIGF ratio is considerably higher than that in plasma (44). While this observation suggests enhanced sensitivity compared with circulating biomarkers, standardization of isolation methods and normalization strategies remains a critical barrier to clinical translation. To summarize, urinary exosomes represent a promising but still evolving platform for non-invasive assessment of renal pathophysiology; future studies should prioritize mechanistic validation, longitudinal designs, and methodological harmonization to substantiate their clinical utility.

### 3.3 Mesenchymal stem cell exosomes

Mesenchymal stem cell-derived exosomes (MSC-Exos) primarily originate from bone marrow, adipose tissue, and umbilical cord. They exert diverse vascular and immunomodulatory effects via paracrine signaling. MSC-Exos have been shown to ameliorate age-associated endothelial dysfunction, arterial stiffness, and renal structural injury (45). MSC-Exos can also modulate the RAAS and facilitate the conversion of Ang II to Ang- (1–7), therefore attenuating cardiac remodeling and providing sustained cardioprotective effects (46). Nevertheless, the extent to which this pathway contributes to long-term blood pressure regulation *in vivo* is not yet fully established. Furthermore, bone marrow MSC-Exos can efficiently deliver miR-126 to HUVECs, which increases VEGF and Ang-I expression and promotes angiogenesis by targeting PIK3R2 to activate the PI3K/Akt signaling pathway (47).

In models of pulmonary and hypoxia-induced hypertension, MSC-Exos exhibit consistent vasoprotective actions. MSC-Exos carrying the apelin gene in pulmonary hypertension rats can activate the PI3K/AKT/eNOS and ERK1/2 pathways, observably reducing pulmonary arterial pressure and mitigating vascular remodeling (48). MSC-Exos also suppress the IL-33/ST2 axis and drive macrophage polarization toward the M2 phenotype, which alleviates inflammation, prevents endothelial apoptosis, and inhibits abnormal proliferation of pulmonary arterial smooth muscle cells (PASMCs) (49). Moreover, they downregulate YAP1 and its downstream target SPP1, attenuating PASMC migration and pulmonary vascular remodeling (50). Additional studies report that MSC-Exos regulate miR-17 and miR-204 by inhibiting Hsp90aa1/ERK/pERK or STAT3 signaling. This regulation improves right ventricular hypertrophy and pulmonary vascular remodeling (51, 52).

The cardioprotective properties of MSC-Exos also involve immune modulation and energy metabolism. Through the S1P/S1PR1 axis, MSC-Exos can enhance M2 macrophage polarization and suppress NF-κB and TGF-β1 signaling, producing anti-inflammatory and anti-fibrotic effects (53). They also increase ATP and NADH levels in cardiomyocytes and mitigate oxidative stress via Akt/GSK-3β activation and c-JNK inhibition (54). Tadalafil preconditioning enriches MSC-Exos with miR-29a-3p, which targets the CREB1-mediated ENPP2 axis, further attenuating hypoxia-induced pulmonary hypertension (55). In preeclampsia models, MSC-Exos can enhance endothelial cells proliferation, migration, and angiogenesis, and also improve gestational hypertension-associated vascular injury through immunomodulatory, anti-inflammatory, and antioxidative mechanisms (56).

In conclusion, MSC-Exos exert multi-layered protective effects spanning RAAS modulation, vascular remodeling, immune regulation, and metabolic homeostasis. MSC-Exos may represent a promising adjunctive strategy for targeting complex, multi-pathway dysregulation in hypertension. While a substantial body of evidence supports their vasoprotective and cardioprotective roles, these findings are predominantly derived from preclinical models, particularly in pulmonary hypertension and hypoxia-related conditions. Thus, their applicability to systemic hypertension remains to be further substantiated.

## 4 Therapeutic prospects of exosomes in hypertension

Exosomes represent a transformative drug delivery platform for hypertension due to their superior biocompatibility, low immunogenicity, and innate capacity to traverse biological barriers. Through endogenous or exogenous engineering, exosomes can encapsulate miRNAs, siRNAs, proteins, or small-molecule drugs and be directed to endothelial cells, VSMCs, and renal cells, offering new therapeutic strategies to counter vascular remodeling, inflammation, and abnormal renal sodium handling.

### 4.1 Endogenous engineering

Endogenous engineering modifies exosome producing cells genetically or pharmacologically, allowing target molecules to be packaged during exosome biogenesis while preserving membrane integrity (66). This approach can enhance delivery specificity and therapeutic efficacy, but its complexity and potential off-target effects *in vivo* require careful validation. Genetic modification can effectively enhance the vascular reparative potential of MSC-Exos. For example, mesenchymal stem cells transduced with lentiviral vectors overexpressing HIF-1α secrete exosomes that, under hypoxic conditions, upregulate VEGF, Ang-1, and PDGF in human umbilical vein endothelial cells, thus restoring migratory, proliferative, and angiogenic capacities (67, 68). Furthermore, hypoxia-induced and glucuronic acid–modified exosomes can accumulate in pulmonary arterial smooth muscle cells with high levels of glucose transporter 1. These exosomes can suppress smooth muscle cell proliferation and ameliorate vascular remodeling through miR-5119-induced calcium signaling (69).

### 4.2 Exogenous engineering

Exogenous engineering loads miRNAs, siRNAs, or drugs into exosomes using methods such as electroporation, sonication, freeze–thaw cycles, or chemical transfection (70). However, electroporation may compromise exosome integrity, and chemical transfection can introduce contaminants. Wang et al. used electroporation miR-320d and miR-423-5p inhibitors into endothelial cell–derived vesicles. In spontaneously hypertensive rats, this treatment significantly improved vascular compliance, suppressed the phenotypic switch of VSMCs toward a synthetic state, and attenuated vascular remodeling (71). Surface modification with targeting ligands or specific peptides are applied to enhance selective delivery to diseased vasculature or cardiac tissue. Kim et al. further developed an engineered exosome delivery system targeting pulmonary hypertension by displaying apelin-13 on IFITM3 scaffold and incorporating a CAR peptide (72). This system accumulated efficiently in diseased lung tissue, markedly reduced pulmonary arterial pressure, vascular remodeling, and ventricular hypertrophy in various pulmonary hypertension models, and showed a favorable safety profile. While these results are encouraging, the therapeutic efficacy in systemic hypertension and the long-term safety of repeated exosome administration in humans have yet to be established.

## 5 Discussion

Exosomes have emerged as critical mediators in the pathogenesis and potential treatment of hypertension. In this review, we synthesize evidence from circulating, urinary, and mesenchymal stem cell–derived exosomes to suggest that exosomal signaling coordinates vascular dysfunction, immune activation, renal sodium handling, and neurohumoral regulation across multiple organ systems. Exosome-derived miRNAs appear to influence several interrelated processes, including impaired endothelial nitric oxide signaling, immune–inflammatory amplification, VSMC phenotypic switching, and RAAS dysregulation. These observations position exosomes as active regulators that may link local vascular injury with systemic blood pressure control.

Nevertheless, interpreting the current evidence requires caution. First, much of the mechanistic insight, such as exosome-mediated RAAS activation in VSMCs the reflection of renal sodium handling in urinary exosomes, originates from animal or *in vitro* studies, and its relevance to human essential hypertension remains to be confirmed. In addition, although multiple miRNAs have been implicated, existing studies tend to adopt a reductionist approach centered on individual cargo molecules. This focus may not fully capture the complexity of exosomal signaling, which in turn limits the reproducibility of proposed biomarker signatures.

Translationally, exosomes are attractive as therapeutic vehicles owing to their biocompatibility and inherent targeting properties. Nonetheless, their biological heterogeneity, stemming from variations in cellular origin and cargo composition, complicates both mechanistic interpretation and the development of standardized therapeutic products (73). Further confounding the literature, variability in isolation methods used across studies, including ultracentrifugation and precipitation-based approaches, affects both yield and purity and contributes to inconsistent results, particularly in biomarker discovery (74). The absence of unified normalization strategies further constrains cross-study comparisons.

The efficiency of exosome-mediated is another important constraint. The low miRNA copy number per vesicle and inefficient cellular uptake can limit functional transfer, and endosomal–lysosomal degradation further diminishes bioavailability (75). These limitations are especially pertinent for engineered exosome therapies, for which preclinical efficacy may not reliably predict clinical outcomes. A further translational hurdle is the incomplete knowledge of exosome pharmacokinetics and biodistribution (76), which hampers dose optimization and long-term safety assessment in a chronic condition like hypertension. Moreover, although exosomal signaling intersects with the RAAS, its potential interplay with conventional antihypertensive therapies has received little systematic investigation.

Future efforts may focus on engineering surface proteins to facilitate endosomal escape, optimizing RNA secondary structures, and improving vesicle manufacturing processes (77). Parallel efforts to establish standardized protocols for exosome isolation and characterization will be essential to enhance reproducibility across studies.

Exosomes should be regarded not merely as passive carriers of molecular cargo, but as active participants in intercellular communication that link vascular, immune, renal, and neurohumoral compartments. This review draws connections among endothelial dysfunction, immune–inflammatory amplification, VSMC phenotypic switching, and RAAS dysregulation—processes that exosomal signaling appears to integrate in the pathogenesis of hypertension. An integrated view of these interactions may help reconcile disparate observations and inform more targeted approaches to diagnosis and treatment.

In summary, exosomes constitute a promising but still maturing area of hypertension research. Successful clinical translation will depend on rigorous validation of mechanisms in human contexts, methodological harmonization, and careful clinical assessment.

## 6 Conclusion

Exosomes are critically involved in the pathogenesis and progression of hypertension by regulating endothelial function, vascular remodeling, renal ion transport, and inflammatory responses. Circulating and urinary exosomes have emerged as biomarkers, while MSC-derived and engineered exosomes represent potential therapeutic strategies. However, their application is limited by biological heterogeneity, lack of standardized isolation and characterization methods, and insufficient clinical evidence. Future studies should prioritize cell type –specific signaling mechanisms, improve isolation and delivery techniques, and systematically evaluate long-term safety and efficacy. Overall, exosomes constitute a promising framework for mechanistic investigation and targeted therapy in hypertension, although further validation is required prior to clinical implementation.
